# The value of FeNO measurement in childhood asthma: uncertainties and perspectives

**DOI:** 10.1186/2049-6958-8-50

**Published:** 2013-07-31

**Authors:** Giuliana Ferrante, Velia Malizia, Roberta Antona, Giovanni Corsello, Stefania La Grutta

**Affiliations:** 1 Institute of Biomedicine and Molecolar Immunology, National Research Council, Palermo, Italy; 2 Department for Health Promotion and Mother and Child, Università di Palermo, Italy

## Abstract

Asthma is considered an heterogeneous disease, requiring multiple biomarkers for diagnosis and management. Fractional exhaled nitric oxide in exhaled breath (FeNO) was the first useful non-invasive marker of airway inflammation in asthma and still is the most widely used. The non-invasive nature and the relatively easy use of FeNO technique make it an interesting tool to monitor airway inflammation and rationalize corticosteroid therapy in asthmatic patients, together with the traditional clinical tools (history, physical examination and lung function tests), even if some controversies have been published regarding the use of FeNO to support the management of asthma in children. The problem of multiple confounding factors and overlap between healthy and asthmatic populations preclude the routine application of FeNO reference values in clinical practice and suggest that it would be better to consider an individual “best”, taking into account the context in which the measurement is obtained and the clinical history of the patient. Besides, there is still disagreement about the role of FeNO as a marker of asthma control, due to the complexity of balance among the different items involved in its determination and the lack of homogeneity in the population groups studied in the few studies conducted so far. Heterogeneity of problematic severe asthma greatly limits utility of FeNO alone as a biomarker of inflammation to optimize the disease management on an individual basis. None of the studies conducted so far demonstrated that the use of FeNO was better than current asthma guidelines in controlling asthma exacerbations. In summary, there is a large variation in FeNO levels between individuals, which may reflect the natural heterogeneity in baseline epithelial nitric oxide synthase activity and/or the contribution of other noneosinophilic factors to epithelial nitric oxide synthase activity. FeNO is a promising biomarker, but at present some limits are highlighted. We would recommend that further research can be carried out by organizing studies aimed to obtain reliable reference values of FeNO and in order to better interpret FeNO measurements in clinical settings, taking also into account the influence of genetic and environmental factors.

## Review

### Introduction

Asthma is a chronic inflammatory disorder of the airways, in which underlying structural and functional changes occur [1]. The increased knowledge of the deep interplay between the pathophysiological pathways of chronic airway inflammation and remodelling in asthma has led to consider asthma an heterogeneous disease, requiring information on multiple biomarkers for diagnosis and management [2]. Fractional exhaled nitric oxide in exhaled breath (FeNO) was the first useful non-invasive marker of airway inflammation in asthma and still it is the most widely used [2,3].

The non-invasive nature and the relatively easy use of FeNO technique make it an interesting tool to monitor airway inflammation and rationalize corticosteroid therapy in asthmatic patients, together with the traditional clinical tools (history, physical examination and lung function tests) [4], even if some controversies have been published regarding the use of FeNO to support the management of asthma in children [5].

FeNO levels correlate with eosinophilic counts in induced sputum or bronchoalveolar lavage fluid as well as with eosinophil infiltration of the airways and peripheral eosinophilia, mainly in atopic subjects [3,6]. Correlations were also found with total IgE, serum eosinophil cationic protein (ECP) and the number of positive skin prick tests, whereas there are only weak correlations with spirometric outcomes and clinical measures of asthma [3,7].

Nevertheless, more recent findings about the positive cross-correlation of daily fluctuations in FeNO values with symptom scores may open a new vision of this biomarker use in childhood asthma [8]. Moreover, it has been recently found a linear association between FeNO and bronchial responsiveness in children with and without asthmatic symptoms suggesting the continuity of bronchial inflammation from health to disease and the importance of this measurement together with other clinical end points, such as lung function, symptoms and symptom history [9,10].

The aim of this review is to elucidate what could be the role of FeNO in the assessment of asthma severity and control. We will also look at the perspectives of considering the relation between FeNO measurement and various clinical phenotypes of pediatric asthma.

#### FeNO reference values: an unresolved issue

Normal values of FeNO and feasibility in children have been already assessed.

**Figure 1:**
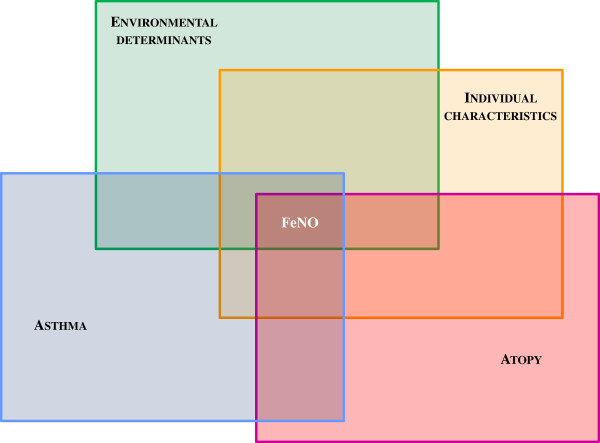
**Disease and non**-**disease**-**related factors influencing FeNO.** FeNO levels in children depend both on non-disease-related factors, such as environmental determinants and clinical characteristics, and disease-related factors, such as asthma and atopy. All these confounding factors have to be considered in clinical setting, because they may influence FeNO values and, consequently, patient’s management.

FeNO levels in healthy children are below 15 to 25 ppb, depending on several non-disease-related factors, such as: age, gender, height, ethnicity, genetics, self-reported atopy, allergic sensitization, total IgE, time of testing, infections, a nitrate rich diet, exercise, smoking, ambient nitric oxide, time of the day and season and environmental pollution [3,11-14] (Figure 1). All these confounding factors have to be considered when evaluating FeNO levels in clinical setting, because they may influence FeNO values and, consequently, patient’s management [6,15].

Studies aimed to define the right cut-point to diagnose asthma using FeNO pointed to a range from 20 to 25 ppb, even if these values may range from 22 to 44 ppb in subjects with well-controlled asthma. This suggests that there is an overlap between mean FeNO levels in healthy and asthmatic people [4].

There is also evidence that atopy, mainly allergic sensitization to perennial allergens, is *per se* able to significantly affect FeNO levels playing an important role in elevating nitric oxide (NO) production, even in asymptomatic subjects, probably through long-lasting inflammatory stimuli in the airways. In our experience, a significant relation between FeNO values and number of positive skin tests was found, with the highest FeNO levels observed in atopic children with doctor-diagnosed asthma. This suggests that atopy and asthma may be the most consistent predictor of high FeNO levels [7].

The problem of multiple confounding factors and overlap between healthy and asthmatic populations preclude the routine application of FeNO reference values in clinical practice and suggests that it would be better to consider an individual “best”, taking into account the context in which the measurement is obtained and the clinical history of the patient [4]. In fact, FeNO values are highly reproducible in an individual, so it might be more successful to use personalized cut-off values for each subject, rather than using a single cut-off value for all patients [2].

#### Asthma control and FeNO

The goal of asthma treatment is achieving control of symptoms and airway inflammation and maintaining pulmonary function [9].

The need for a reliable method to assess the level of asthma control is related to the recent update of international guidelines, suggesting that tailored asthma treatment to the level of disease control, rather than severity, should be performed [16].

Although the burden of airway inflammation in the clinical phenotypes is still under debate, inflammation has an important pathophysiological role for the management of chronic respiratory diseases and is relevant in guiding pharmacological therapy [17,18]. In this context, FeNO might be an useful tool in evaluating asthma control.

FeNO values are related to several markers of asthma control, such as night time symptoms, beta-agonist use, and bronchodilator reversibility [19], as well as to the use of oral or inhaled steroid treatment [20]. In addition, previous studies showed that FeNO is raised in asthmatic children, especially when asthma is uncontrolled and during exacerbations [15]. Hence, FeNO may provide useful information about airway inflammation as a complementary tool to lung function tests, in order to obtain a better control of asthma symptoms and an optimal rationalization of anti-inflammatory therapy.

Conversely, recent studies found that a treatment aimed at lowering FeNO levels in asthmatic children cannot improve clinical markers of asthma control [21], and inconsistent data were found about the correlations between FeNO and Asthma Control Test scores, both in adults and in children [9]. So, there is generally no evidence that the measurement of FeNO adds value as a predictor of asthma control compared to conventional tests of lung function or that regular measurement of FeNO leads to important benefits in adjusting the dose of steroid therapy, thus this value alone cannot be recommended to assess asthma control [4]. However, it should be noted that the disagreement between FeNO and lung function is probably due to the lack of homogeneity of the different population groups of children studied, with different inclusion criteria, concerning the severity of asthma, the use of inhaled corticosteroid therapy and dosage, presence of atopy, as well as different definitions of control used [5,22] (Table 1).

**Table 1:** Asthma control and FeNO

**Author**	**Age range(years)**	**Subjects (number)**	**Correlation**	**Direction of effect**
**Covar RA, JPediatr 2003**	**5–12**	**92 with mild to moderate asthma**	**. degree of bronchial hyperresponsiveness**	**Yes**
			**. bronchodilator reversibility**	
			**. allergen skin prick tests**	
			**. serum IgE**	
			**. eosinophil count**	
			**.nocturnal symptoms**, **β-agonist use at least once weekly**.	
**Smith AD, J Allergy ClinImmunol 2009**	**12–75**	**73 with chronic asthma in treatment with ICS**	**. asthma control (changes in symptoms, bronchodilator use, diurnal peak flows, spirometry)**	**No**
**Fritsch M, Pediatr Pulmonol 2006**	**6–18**	**47 with mild to moderate persistent asthma**	**. dose of ICS(p < 0.002)**	**Yes**
			**. b**-**agonist use 2 weeks prior to a visit(p<0.05)**	
			**. asthma symptoms(p < 0.0001)**	
			**. bronchial hyperresponsiveness(p = 0.02)**	
**Waibel V, Pediatr Pulmonol 2012**	**12(mean age)**	**107 with a diagnosis of asthma**	**. C**-**ACT**	**No**
**Cabral ALB, AnnalsAllergy Asthma Immunol 2009**	**7–14**	**32 with moderate-to-severe asthma**	**. risk for exacerbations**	**No**
**Green RJ, Chest 2013**	**4–11**	**71 with atopic asthma**	**. spirometry**	**No**
			**. ACT**	

**Figure 2:**
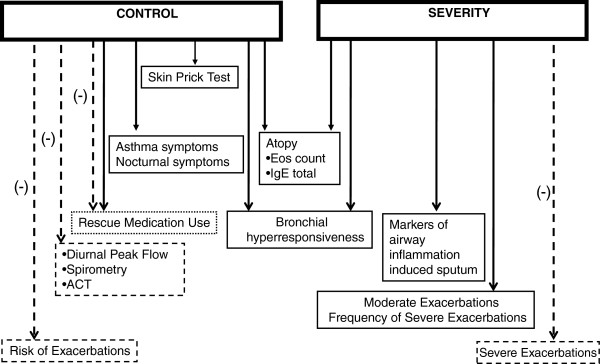
**Role of FeNO as a marker of asthma control and severity.** The continuous lines indicate the evidence of relationship between FeNO and several markers of asthma controls; the dashed lines indicate no evidence.

In summary, there is still disagreement about the role of FeNO as a marker of asthma control, due to the complexity of balance among the different items involved in its determination and the lack of homogeneity in the population groups studied in the few studies conducted so far (Figure 2). Because of the complex nature of the disease, asthma control reasonably needs more than one tool in assessment and both physician evaluation and objective testing are required [5].

#### Severity of asthma and FeNO

Although the majority of children with asthma respond well to standard therapy, a significant proportion still have problematic, severe disease that is not controlled with conventional management.

The utility of FeNO for monitoring children with moderate-to-severe asthma is uncertain. In fact, studies aimed to evaluate FeNO usefulness as a predictor of asthma exacerbations show conflicting results. Moreover, there is not consensus yet about the optimal FeNO cut-point level to define high risk of exacerbation. A recent study by Cabral et al. showed no benefits in tapering ICS doses in atopic children by monitoring FeNO levels, suggesting that this tool has a limited value as a predictor of asthma exacerbations [22]. Conversely, some data reported that FeNO might be helpful in predicting and preventing exacerbations. Gagliardo et al. found a significant correlation between FeNO levels and other markers of inflammation, such as sputum eosinophilia and IL-8, and the number of severe exacerbations in asthmatic children [23]. More recently, Van der Valk et al. studied longitudinal daily FeNO measurements in relation to exacerbations in atopic asthmatic children founding changes in FeNO prior to moderate, but not severe, exacerbations. The Authors speculated that moderate exacerbations were probably preceded by increased eosinophilic airway inflammation and that the level of cross-correlation between FeNO levels and symptoms could identify children at risk for exacerbations. However the study sample size was small and the therapeutic intervention with inhaled corticosteroid (ICS) could have modified the association between FeNO and exacerbations [24] (Table 2).

**Table 2:** Asthma severity and FeNO

**Author**	**Age range(years)**	**Subjects (number)**	**Correlation**	**Direction of effect**
**Gagliardo R, PediatrAllergyImmunol 2009**	**6**-**14**	**35 with asthma**	**. eosinophilia in induced sputum**	**Yes**
			**. IL**-**8 in induced sputum**	
			**. allergen skin prick tests**	
			**. number of severe exacerbations**	
**Van der Valk RJ, Allergy 2012**	**10**.**9(mean age)**	**72 with asthma**	**. moderate exacerbations**	**Yes**
**Franklin PJ, Thorax 2003**	**11**.**5(mean age)**	**155 enrolled from an unselected population**	**. atopy (p < 0.001)**	**Yes**
			**. level of AR (p = 0.005)**	
			**. blood eosinophil count (p = 0.007)**	
			**. bronchial hyperresponsiveness (p = 0.02)**	

*AR* airway reactivity.

Some factors such as genetic variation in FeNO levels, increased diffusion of NO in asthmatic airways, the association of FeNO with atopy and airway hyperresponsiveness, may have weight on child’s asthma difficult to control leading to higher FeNO levels [25]. Moreover, it has been emphasized that many children may have factors apart from the underlying severity of asthma that contribute to their severe disease, including comorbidities, socioeconomic problems, adverse environmental exposures (such as tobacco smoke, relevant allergens and other harmful factors), psychological problems and poor adherence to treatment.

Hence, heterogeneity of problematic severe asthma greatly limits utility of FeNO alone as a biomarker of inflammation to optimize the disease management on an individual basis.

#### Management of asthma and FeNO

There is a strong interest to use FeNO as a guide for asthma treatment, based on the premise that FeNO reflects airway inflammation. In fact, in some studies FeNO is validated as a useful tool both in diagnosing and managing patients with atopic asthma [26].

Sensitivity and specificity of FeNO measurements were showed to be acceptable to discriminate asthma from other non-asthmatic conditions in previous clinical studies. However, it should be remembered that normal FeNO levels do not exclude the diagnosis of asthma, especially in non atopic subjects. On the other hand, elevated values, while suggesting asthma, might be insufficient for the diagnosis and management of the disease, mainly in clinically controlled patients [27]. Thus, the question is whether to treat patients according to the clinical control and lung function tests or according to the FeNO values, which may suggest a latent inflammation [26].

It has been suggested that FeNO may be more appropriate for tapering, rather than for stepping up anti-inflammatory treatment and could be used mainly as an indicator of the patient’s compliance with the prescribed therapy [3]. In addition, the relatively rapid change of FeNO levels after steroid treatment suggests its utility in monitoring adherence to and response to therapy, being an indicator of patient compliance with the prescribed therapy [2,4].

Nevertheless, two meta-analysis of paediatric studies showed that FeNO monitoring lead to increased use of ICS, without significant influence on lung function outcomes (FEV_1_) compared to conventional management [28,29]. In fact, while two studies have failed to observe any improvement in asthma control compared with the use of standard asthma guidelines, even using a daily FeNO monitoring [30,31], other two studies showed an increased ICS dose in FeNO follow up group compared to conventional follow up group with no changes in asthma control [21]. In summary, none of these studies was able to demonstrate that the use of FeNO was better than current asthma guidelines in controlling asthma exacerbations. Moreover, the data suggest that using FeNO to tailor the dose of ICS cannot be recommended in routine clinical practice, because of the danger of excessive doses without significant changes in clinical outcomes. High FeNO levels may be caused by non-disease related factors that clinicians should be aware of in asthma management. To date, a guideline-based approach still remains essential [28].

In 2011, the American Thoracic Society (ATS) guidelines suggested that decision cut-points rather than reference values have to be used in FeNO levels interpretation. Specifically, the guidelines stated FeNO values < 25 ppb (20 ppb in children <12 years) a low likelihood of eosinophilic inflammation and corticosteroid response, while FeNO values >50 ppb (35 ppb in children <12 years) indicate otherwise [4]. Similarly, a recent work by See et al. showed that in the US general population there is a large variation of normal FeNO levels; values >39 ppb in subjects aged 12 to 80 years (36 ppb in children aged 6–11 years) indicated abnormality and a high risk of airway inflammation [32]. These data support the ATS guideline recommendations that clinical interpretation of FeNO should depend on thresholds rather than reference values. However, further studies are needed to get more reliable FeNO cut-off values for treatment decisions [28].

#### Asthma phenotypes and FeNO

Asthma is a complex disease characterized by different underlying pathophysiologies. This biological heterogeneity translates into clinical practice in different phenotypes of the disease. Clinical phenotypes of asthma are subgrouped according to characteristics such as symptoms, inflammation, lung function and treatment response. Recently, much effort has been done to link this features with molecular pathways defining the new concept of ‘endotype’. This term refers to the patient’s characterization including natural history, genetics and clinical features, arising from an underlying specific pathobiology associated with reliable biomarkers and a predictable response to therapy. The identification of this subgroup of patients is fundamental to design a targeted treatment strategy and improve treatment response, achieving the control of the disease. Originally asthma was classified in extrinsic (allergic) and intrinsic (non allergic) asthma. Most recently other asthma phenotypes have been identified, including early onset allergic asthma, exercise induced asthma and non Th2-associated asthma (neutrophilic asthma, obesity-related asthma and smoking asthma) [33].

Elevated levels of FeNO are reported as a biomarker for an allergic asthma phenotype. Several studies highlighted the relationship between FeNO and eosinophilic airway inflammation [4]. Moreover, various studies demonstrated that FeNO is increased in atopic individuals with and without asthma, suggesting that atopy and asthma could be cofactors in determining elevated FeNO levels [7,34,35].

Recently, it has been shown that baseline FeNO levels are high in children with exercise-induced bronchoconstriction and correlate with the degree of post-exercise bronchoconstriction, suggesting that FeNO may be a predictor of airway hyperresponsiveness to exercise, particularly in asthmatic children sensitive to indoor allergens [36].

Many studies evaluated the relationship between FeNO and obesity in asthmatic patients showing no significant relationship between FeNO and Body Mass Index in asthmatic subjects, and suggesting that childhood obesity is not associated with increased airway inflammation [7,11,37,38].

Finally, there is evidence that active smoking and cigarette smoke exposure are correlated with lower FeNO levels both in adults and in children with asthma. This could be due to different mechanisms according to the type of exposure. Acute exposure induces a marked but transient decrease in FeNO levels related to a negative feedback of inducible NO synthase (iNOS) activity, since tobacco smoke contains high concentrations of nitric oxide. Daily smoke exposure is probably associated with a progressive negative feedback leading to the inhibition of iNOS gene expression [6].

Despite this evidence, a recent work by Mahut et al. found that FeNO is not associated with a clinically relevant phenotype in asthmatic children, in accordance with other trials failing to demonstrate the clinical usefulness of this measure for asthma control [39].

In summary, there is a large variation in FeNO levels among individuals, which may reflect the natural heterogeneity in baseline epithelial nitric oxide synthase activity and/or the contribution of other noneosinophilic factors to epithelial nitric oxide synthase activity. Interindividual variation in FeNO combined with the inherent heterogeneity of asthma increases the background noise, which renders FeNO a relatively insensitive tool for guiding therapy in all asthmatics [40]. However, considering the relationship between eosinophilicinflammation and steroid responsiveness in airway disease, it is plausible to use FeNO as an indicator of treatment response. Since not all asthmatic patients respond to ICS therapy, FeNO might help to identify patients with asthma-like symptoms who could benefit or not from corticosteroid treatment [4].

## Conclusions

The development of a diagnostic tool for asthma at an early age, based on non-invasive inflammatory biomarkers in exhaled breath should be a milestone for the good asthma diagnosis and management in childhood. In this context, the data of literature indicate that the assessment of non-invasive markers, such as FeNO in asthmatics, would be an additional field for the best detection of inflammatory components.

Actually, FeNO assessment shows some advantages, including the good correlation with symptoms of asthma, use of beta-agonist and risk of exacerbations. Since FeNO as a marker of eosinophilic airway inflammation has the potential to serve as an indicator of the adequacy of ICS-anti-inflammatory treatment, it could be successfully used to monitor the response to ICS-therapy, improving the tailored steroid therapy in asthmatic children for a better control of the disease [15]. Lastly, FeNO assessment might be useful to identify patients with uncontrolled diseases and to verify the usefulness of new therapeutic approaches [41].

Considering the prevalence of childhood asthma and its associated burden, it is mandatory to obtain an optimal control of the disease, improving outcomes for patients as well as reducing costs attributable to poorly controlled patients [42]. Currently, an economic evaluation of FeNO measurement is scanty, even if Berg and coworkers showed that using FeNO in asthma diagnosis and management in Germany is less costly than management based on standard guidelines; in fact the FeNO assessment cost was offset by the reduction of exacerbations and hospitalizations, due to a better control of the disease, especially in mild to severe adult patients [43]. Furthermore, a study conducted in the United Kingdom showed similar findings in adults, suggesting that FeNO measurement can be considered a cost-effective alternative to standard tests for both asthma diagnosis and management as well as an useful tool in providing a more complete picture of airway status [44]. On the contrary, the results from clinical trial in asthmatic children show that FeNO assessment in asthma management is more expensive and does not have predictive value [45].

Although FeNO is a promising biomarker, at present some limits are highlighted. Essentially, some disagreement exists about the appropriate cut-off points of normal FeNO level, which is crucial to guide the appropriate clinical response. As previously discussed, its utility is lost when it is applied to all asthmatics because of the underlying heterogeneity of the phenotypes.

In practice, before considering biomarkers for a clinical use, we must appreciate that asthma is a heterogeneous disease and a panel of biomarkers is needed to indicate the various and different underlying disease pathologies. Regardless of the complexity or completeness of a future panel of asthma biomarkers, it is highly unlikely that they will completely replace pulmonary function testing in clinical practice. Indeed, biomarker testing will be designed to complement rather than replace existing methods of clinical diagnosis and disease monitoring.

### Towards future research

In light of the reviewed evidence, we would recommend that further research can be carried out by organizing studies aimed to obtain reliable reference values of FeNO, taking also in account the influence of genetic and environmental factors. Moreover, further research is required to better interpret FeNO measurements in clinical settings, as FeNO could be useful in understanding asthmatic patients in whom more than one factor contributes to respiratory symptoms (obesity, anxiety, environmental exposure). Finally, more studies are needed to understand the role of FeNO in monitoring response to therapy [4]. Therefore, a periodically update of specific guidelines relating to this rapidly evolving field of research is strongly desirable.

## Competing interests

Financial competing interests

• In the past five years the authors have not received reimbursements, fees, funding, or salary from an organization that may in any way gain or lose financially from the publication of this manuscript, either now or in the future.

• The authors do not hold any stocks or shares in an organization that may in any way gain or lose financially from the publication of this manuscript, either now or in the future.

• The authors not currently applying for any patents relating to the content of the manuscript. The Authors have not received reimbursements, fees, funding, or salary from an organization that holds or has applied for patents relating to the content of the manuscript.

• The authors do not have any other financial competing interests.

Non-financial competing interests

There are not non-financial competing interests (political, personal, religious, ideological, academic, intellectual, commercial or any other) to declare in relation to this manuscript.
